# Poor prognostic factors of femoral shaft fractures in children treated by elastic intramedullary nailing

**DOI:** 10.1051/sicotj/2020031

**Published:** 2020-08-31

**Authors:** Alexandru Ulici, Elena Odagiu, Oana Haram, Adelina Ionescu, Gabriel Alin Sterian, Madalina Carp, Iulia Tevanov

**Affiliations:** 1 Department of Pediatric Orthopedic Surgery, Emergency Hospital for Children “Grigore Alexandrescu” 30-32 Iancu de Hunedoara Blvd. 011733 Bucharest Romania; 2 “Carol Davila” University of Medicine and Pharmacy Bulevardul Eroii Sanitari 8 București 050474 Romania

**Keywords:** Femoral shaft fracture, Flexible intramedullary nail, Pediatric trauma, Pediatric orthopedics

## Abstract

*Introduction*: Femoral shaft fractures in pediatric patients are treated by elastic intramedullary nailing using titanium or stainless-steel nails. The elastic stable intramedullary nailing behaves as an internal splint, promoting early mobilization. This type of treatment involves a minimally invasive approach, no damage to the growth plates, and no impairment of femoral head blood supply. *Purpose*: The aim of our study was to identify the negative predicting factors that might lead to an increased complication rate after elastic stable intramedullary nailing of femoral shaft fractures in children. *Methods*: We conducted a retrospective study on 137 patients with femoral shaft fractures treated by elastic stable intramedullary nailing. Patients’ age ranged between 4 and 17 years. We used data from the medical records of the patients to evaluate postoperative complications. Plain radiographs were analyzed to determine the fracture type, fracture location, and postoperative complications such as delayed union, angular deformities, and limb length discrepancies. Multivariate analysis was conducted to identify predictors for poor outcomes. *Results*: Complications occurred in 29 patients (21%) and consisted of delayed union, axial deformities, or lower limb length discrepancies. In the group of patients that suffered from complications, mechanism of injury, age, and weight were significant. They were older by an average of 5 years; half of them weighed more than 50 kg and over a half were involved in a road traffic accident. *Conclusions*: Elastic nailing is a successful tool to treat femoral shaft fractures. Three factors were demonstrated to influence the outcome. The mechanism of injury, age > 11 years, and weight > 50 kg are the most important and are predictors for development of complications such as delayed union or deformity.

## Introduction

Femoral shaft fractures represent 2% of all pediatric fractures and have a bimodal distribution based on patient’s age [1]. Thus, in childhood, the femoral diaphysis is relatively weak and it could break during casual activities (childhood games), while throughout adolescence, 90% of the fractures are secondary to high energy trauma, such as motor vehicle accidents [2]. There are several factors that may influence the treatment of femoral shaft fractures including the age and weight of the child, associated lesions, type of fracture, surgeon’s preferences, and socioeconomic status [1, 2].

Closed reduction and spica cast immobilization is the best treatment for femoral shaft fractures in children aged less than 4 years [3]. In older patients, the use of different types of fixation is needed [4].

Fractures heal following the same stages in both children and adults; fractures in children are particular due to the osteogenic status of the pediatric bone and the healing process that is already ongoing when the bone fractures, while in adults the bone healing factors must be stimulated [5].

Elastic stable intramedullary nailing (ESIN) has become one of the main treatments for femoral shaft fractures in pediatric population. The elasticity of the nails allows the formation of external callus through micromovements at the fracture site [6]. This treatment does not have an impact on the growth plates and allows early mobilization [7]. Additionally, it has esthetic postoperative scars, facile removal, shortening of the inpatient period, good cost-effectiveness ratio, and less psychological impact on the patient [7, 8].

The insertion of the elastic nails in an antegrade or retrograde manner is influenced by the site of the fracture [9]. Fricka showed that the retrograde double-C shaped design of the nails provides greater resistance to deformation than antegrade C and S shaped model [9].

Lascombes et al. observed a low incidence of postoperative complications when the nails filled 80% of the medullary canal [10]. The French School emphasizes on the importance of nail contouring before insertion, so that the apex of the nail is at the fracture site [11]. The two nails must provide mutual support, preventing deformation by bending and ensuring rotational control [11].

Elastic nails do not provide rigid fixation at the fracture site [10, 12]. Although this is beneficial in many children fractures, it has drawbacks in unstable fractures such as long spiral and comminuted fractures. These may shorten and angulate, mostly in overweight patients (>45 kg) and children who are older than 11 years of age [12, 13].

The complications following the ESIN treatment are relatively rare [14]. According to the literature, the most common complication is irritations at the protruded ends of the nail. This irritation may cause soft tissue and intraosseous infection. Other complications have been reported including nonunion, malunion with slight varus or valgus deformity, and limb length discrepancy. Recent studies show that an overgrowth of 1 cm occurs in 8.2% of preschool children [15]. Özdemir et al. analyzed limb length discrepancies and discovered an average difference of 1.8 mm, with no clinical significance [16].

A few studies reported on poor prognostic factors that predict complications after ESIN [12, 13]. The aim of our study was to validate these studies and also to identify other potential poor predicting factors that might lead to an increased complication rate.

## Materials and method

With the approval of the hospital’s ethics committee (registrations number 8154), we conducted a retrospective study on 137 patients diagnosed with femoral shaft fracture who underwent close reduction and internal fixation using two elastic nails. The patients were admitted to our clinic between the 1st of January 2014 and the 31st of January 2018.

Our study included pediatric patients with femoral shaft fractures, aged between 4 and 17 years. We excluded pathological bone fractures (benign bone tumors, systemic diseases associated with bone fragility such as Osteogenesis Imperfecta etc.), open fractures, fractures that were treated by cast immobilization only, by open reduction and internal fixation, and other types of osteosynthesis such as internal fixation using plate and screws, external fixation, interlocking nail, etc.

We analyzed the patient’s demographic data (age, gender, home environment, weight), side of injury (left/right), the location and type of fracture, and the occurrence of postoperative complications. Standard frontal and lateral view radiographs were evaluated to assess the stability of the fracture. Transverse and short oblique fractures are considered as stable fractures whereas long spiral and comminuted fractures are considered unstable. The site of fracture is grouped into proximal third, middle third, or distal third. The follow-up period was 12 months.

All the surgical procedures were performed by pediatric orthopedic surgeons under general anesthesia. After adequate closed reduction of the fracture, two titanium or stainless steel elastic nails were introduced. No endcaps were used.

The diameter of the nail varied between 2 and 4.5 mm. The size of the nail was established by multiplying the narrowest diameter of the bone canal by 0.4. The location of the fracture influenced the surgical approach. Thus, retrograde nailing was performed for middle or proximal shaft fractures, while antegrade approach was used for distal third fractures. In case of length unstable fractures, spica cast immobilization was used, for a period of time that ranged between 3 and 5 weeks. Nail removal was performed after a mean period of 10 months.

**Figure 1:**
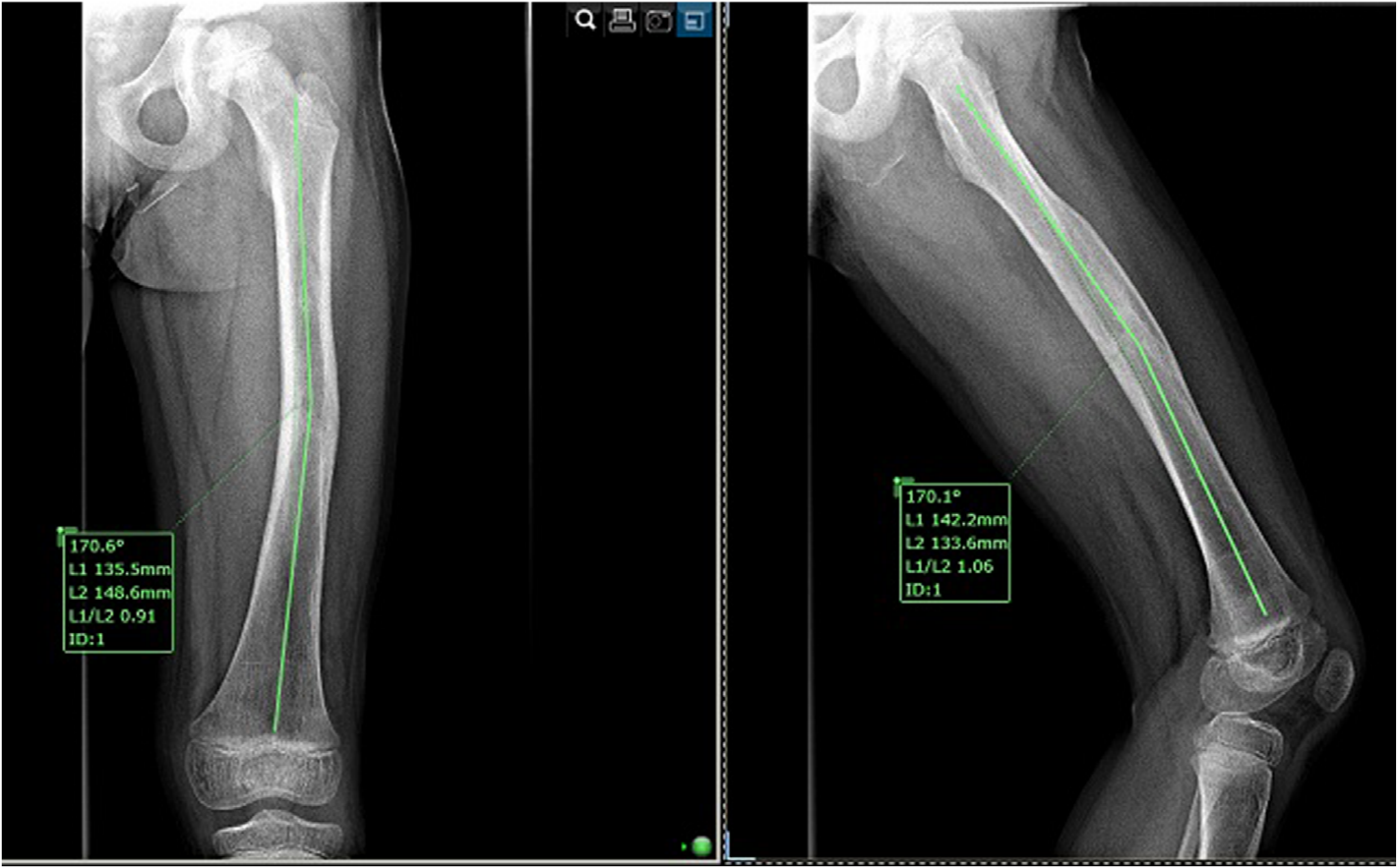
An 11-year-old patient with sagittal deformity (9.4° procurvatum) and coronal deformity (11.2° varus), 18 months after elastic stable intramedullary nailing for a mid-shaft femoral fracture.

The callus formation was evaluated at 6 and 12 months on plain radiographs in standard views using Economedes’ Method [17]; lower limb length discrepancies were determined on scanograms. Angulation deformities were measured on orthogonal radiographs by determining the angle between the anatomical axes of the bone fragments, distal and proximal to the fracture site, as seen in Figure 1.

Patients were divided into two groups: complicated and uncomplicated based on whether they did develop carefully selected complications or not. The selected complications included delayed union and deformities in any plane including leg length discrepancy. The values were compared to literature data of acceptable residual deformities following femoral fractures [18]. Patients with values outside the acceptable ranges are considered complicated.

The data was collected from patient’s medical records and radiographs. Continuous data were recorded as mean, standard deviation (SD), and number of patients to indicate group size, with the treatment effect being reported as the mean difference (MD) with corresponding 95% confidence interval (95% CI). Dichotomous data were expressed as proportions, with the treatment effect reported as a risk ratio (RR) with 95% CI. Statistical significance was set at *P* < 0.05.

Several prognostic factors are considered including qualitative variables such as gender, home environment, fracture mechanism, location and fracture type, fracture stability, and quantitative variables such as age, weight. Based on their weight the patients were divided into two groups: less than 50 kg and over 50 kg because previous studies showed that children who are over 50 kg are at a higher risk of complication [13, 19]. Univariate analysis was performed initially to understand the influence of individual factors on the outcome but generalized logistic regression models were also used to understand the interaction between the prognostic factors themselves. Statistical analysis was performed using the SPSS statistical software.

## Results

The mean age for femoral fractures in our patients was 8 years (range: 4–17 years) with males involved as twice as females. The place of residence was predominantly urban (64%). Forty percent of the fractures involved high energy trauma while others were caused by falls. The most common location of the fracture occurred in the middle third (*n* = 72, 52 %). Seventy percent of the fractures were considered stable and 30% were unstable fractures. Seventy-two percent of the patient weighed less than 50 kg (*n* = 99) whereas 28%, weighed over 50 kg (*n* = 38).

Most of our patients (*n* = 108, 79%) were successfully treated by closed reduction and internal fixation using elastic nails without any complication. This group of patients is categorized as the uncomplicated group. Selected complications (delayed union and deformities) occurred in 29 patients (21%) and this comprises the complicated group.

Univariate analysis shows that site of fractures, fracture stability, and gender of the patient did not influence complication rates. The surgical approach whether antegrade or retrograde or the size of the nail used in relation to the size of the femoral shaft medullary canal did not cause more complications as well. However, three factors were associated with significantly higher complication rates. Children who were over 11 years old and those who weighed more than 50 kg had significantly more complications rates (χ^2^: *P*-value < 0.001). Children whose fractures were caused by road traffic accidents were at a higher risk of complications (χ^2^: *P*-value = 0.008) (Table 1).

**Table 1:** Univariate analysis of the prognostic factors.

Factors	Complicated	Uncomplicated	χ^2^: *P*-value
Mechanism of injury (RTA/non RTA)	16/13	31/77	0.008
Stability (stable/unstable)	11/18	30/78	0.29
Approach (antegrade/retrograde)	9/20	22/86	0.225
Fracture site (proximal/middle/distal)	7/14/8	29/58/21	0.452
Medullary canal filling (<80% / >80%)	25/4	97/11	0.582
Weight (<50 / >50 kg)	10/19	89/19	**<0.001**
Age (under 11/above 11)	10/19	89/19	**<0.001**
Gender (male/female)	17/12	76/32	0.231

*In bold the *P*-values under 0.05.

In our patients, there was a strong association between age and weight with 97% (*n* = 96) of patients who are under the age of 11 years weighing less than 50 kg whereas 92% (*n* = 35) of the children who were over 11 years weighed over 50 kg. This was statistically significant (χ^2^: *P*-value < 0.001). Furthermore, 64% of children who are younger than 11 years old were involved in road traffic accident in comparison to 36%. This did not reach statistical significance (χ^2^: *P*-value = 0.112). Given the interplay between these three important prognostic factors, a multivariate analysis demonstrated that the only significant factor for complication was the mechanism of injury (*P*-value = 0.029) (Table 2).

**Table 2:** Multivariate analysis of the prognostic factors for femoral shaft fracture.

Factors	*B*	*SE*	Wald	*P*-value	Exp(*B*)	95% CI for Exp(*B*)	
						Lower	Upper
Mechanism of injury	1.139	0.521	4.778	0.029	3.124	1.125	8.676
Stability	−0.549	0.538	1.042	0.307	0.577	0.201	1.658
Approach	−1.347	0.970	1.928	0.165	0.260	0.039	1.741
Fracture site	0.414	0.600	0.476	0.490	1.513	0.467	4.904
Medullary canal filling	1.831	1.150	2.534	0.111	6.238	0.655	59.408
Weight	0.885	0.759	1.361	0.243	2.424	0.548	10.726
Age	0.689	1.127	0.374	0.541	1.991	0.219	18.121
Gender	−0.626	0.535	1.369	0.242	0.534	0.187	1.527

## Discussions

The elastic stable intramedullary nailing behaves as an internal splint, promoting an early mobilization of the patient. This type of treatment involves a minimally invasive approach, no damage to the growth plates and no impairment of femoral head blood supply [20]. Over the past 10 years, intramedullary nailing using steel or titanium elastic nails has been promoted in a double C-shaped fashion [11]. To determine the negative prognostic factors and the complication rates of femoral shaft fractures in children treated by ESIN, we performed this retrospective study.

In our study, the univariate analysis showed a statistically significant relationship between age, weight, mechanism of injury, and complication rates. The mean age in the group of patients that suffered complications was 11 years and 2 months, while, the mean age in the group of patients without complications was 6 years. Out of the 29 patients who experienced postoperative complications, 19 were weighting more than 50 kg (65.5%). Fifty-five percent of patients who developed complications had road traffic accident as a cause of their fracture in comparison to 29% only in the uncomplicated group. Moroz and colleagues reported similar findings regarding the weight and age [19]. In their study, they found out that “a poor outcome was five times more likely in children who weighed more than 49 kg” [19]. Sagan et al. determined in their research on 70 fractures treated by ESIN, that a mean weight of 46.5 kg was an important predictive factor of an anterior bow deformity greater than 15° [21].

The new interesting finding of our study is that the multivariate analysis demonstrated that the age and weight were dependent on the mechanism of injury which was more significant than both. As expected, weight and age are correlated with each other. The interplay between these two factors was demonstrated in our study with most patients who are over 11 years weighed more than 50 kg. We anticipated a similar interplay between age and the frequency of road traffic accident with older children being more prone. Although, 64% of children who are younger than 11 years were involved in road traffic accident in comparison to 36%, this did not reach statistical significance. This complex relationship among these important prognostic factors is further explored with multivariate analysis to determine the importance of each to predict outcome. We used a stepwise method for variable selection in logistic regression analysis and we found the mechanism of injury is an important factor, which is clinically plausible.

In the analyzed group, complications such as delay in consolidation, deformation in coronal and sagittal planes, or shortening of the affected limb occurred more often in the distal third femoral shaft fractures, 8 of 21 (27.6%) distal third compared to 14 of 58 (19.4%) in the middle third, but this finding was not statistically significant (*P* = 0.454). Moroz’s study also showed a statistically nonsignificant result; poor outcome was determined in a higher percentage in fractures of the distal third, 6 of 33 (18%), compared to 14 of 164 (9%) in the middle third [19].

Regarding length stability, the fractures in our study were divided into two groups: length stable (transverse and short oblique) and unstable fractures (long oblique and comminuted). Some studies found that length unstable fractures can have a negative outcome because of the associated postoperative complications such as loss of reduction and malunion [22]. In his study on 39 patients, Sink concluded that “the complications that required unplanned surgery for either prominent nails or loss of reduction occurred more commonly in unstable non-transverse fracture patterns” [23]. According to our research, a higher stability of the fracture site does not correlate with lower rates of postoperative complications (*P* = 0.293), compared to unstable fractures. Based on our finding, unstable femur fractures treated by ESIN and spica cast immobilization are not at an increased risk of more complications such as angular deformities, delayed union, limb length discrepancies, or higher premature nail removal rates.

We evaluated the callus formation according to Economedes’ method, where at least three cortical edges are visibly united on the radiographs [17]. The callus formation was checked at 6 months and 12 months on plain radiographs in standard views (frontal and lateral). The results were compared to medical literature. A delayed union was considered when less than three cortical edges were visibly united on the radiograph at the 6-month follow-up; a delayed union that persisted at the 12-month follow-up was defined as a pseudarthrosis or nonunion [19, 21]. In our study, one of the postoperative complications consisted of delayed union (*n* = 13, 9.48%). Seven patients that suffered from delayed union had other associated angular deformities at the 6-month follow-up. These deformities persisted at the 12-month follow-up, but none of the fractures emerged toward a nonunion.

According to the literature, the most common early complications are soft tissue lesions because of nail prominence and nail migration [20]. Knee pain, bursitis, and limitation of the knee flexion could not be evaluated in our research, because there were no recorded data for these symptoms. Several researches encountered more severe complications because of nail prominence such as skin ulcerations and deep wound infections [24, 25]. To prevent these types of complications, endcaps can be used in order to protect the skin from the sharp cutting end of the nail and allow fracture healing without further nail migration and bone telescoping [26]. In our study, there were no cases of skin perforation because of nail prominence that needed a revision procedure or premature nail removal. Endcaps were not used for the patients included in our study.

## Conclusion

Elastic nailing is a successful tool to treat femoral shaft fractures. Majority they do well. Three factors were demonstrated to influence the outcome. The mechanism of injury, age > 11 years, and weight > 50 kg with the mechanism of injury being the most important and the only independent predictor for development of complications such as delayed union or deformity.
